# Global Proteomic Analysis Reveals Inflammatory Pathway Modulation Associated with miR-146a in LPS-Stimulated Macrophages

**DOI:** 10.3390/ijms27146514

**Published:** 2026-07-22

**Authors:** Marut Tangwattanachuleeporn, Aunyamon Srichaimongkol, Jiradej Makjaroen, Sita Virakul, Saharat Nanthawong, H. Sunny Sun, Ni Nyoman Ayu Dewi, Tanapat Palaga, Thidathip Wongsurawat, Asada Leelahavanichkul, Poorichaya Somparn

**Affiliations:** 1 Faculty of Allied Health Sciences, Burapha University, Chon Buri 20130, Thailand; tp.marut@gmail.com; 2 Research Unit for Sensor Innovation, Burapha University, Chon Buri 20130, Thailand; 3 Master of Science Program in Medical Sciences, Faculty of Medicine, Chulalongkorn University, Bangkok 10330, Thailand; aunyamon.s@gmail.com; 4 Center of Excellence in Systems Biology, Faculty of Medicine, Chulalongkorn University, Bangkok 10330, Thailand; saharat.nanthawongb@gmail.com; 5 Department of Transfusion Medicine and Clinical Microbiology, Faculty of Allied Health Sciences, Chulalongkorn University, Bangkok 10330, Thailand; jiradejmak@gmail.com; 6 Department of Microbiology, Faculty of Science, Chulalongkorn University, Bangkok 10330, Thailand; sita.v@chula.ac.th (S.V.); tanapat.p@chula.ac.th (T.P.); 7 Institute of Molecular Medicine, College of Medicine, National Cheng Kung University, Tainan 70101, Taiwan; hssun@mail.ncku.edu.tw; 8 Department of Biochemistry, Faculty of Medicine, Udayana University, Badung 80361, Indonesia; ayu.dewi@unud.ac.id; 9 Division of Medical Bioinformatics, Research Department, Faculty of Medicine, Siriraj Hospital, Mahidol University, Bangkok 10700, Thailand; thidathip.won@mahidol.edu; 10 Center of Excellence in Translational Research in Inflammation and Immunology (CETRII), Faculty of Medicine, Chulalongkorn University, Bangkok 10330, Thailand; aleelahavanit@gmail.com

**Keywords:** inflammation, miR-146a, proteomics, nitric oxide, lipopolysaccharide

## Abstract

Inflammation is essential for host defense, but, when dysregulated, it contributes to tissue damage and chronic disease. MicroRNA-146a (miR-146a) is a well-recognized negative regulator of inflammatory signaling, primarily through suppression of the NF-κB pathway; however, its broader proteomic impact under inflammatory conditions remains incompletely defined. In this study, we overexpressed an miR-146a mimic in lipopolysaccharide (LPS)-stimulated RAW 264.7 macrophages and applied quantitative mass spectrometry to characterize global protein abundance changes. Functional overexpression was supported by reduced mRNA abundance of the established miR-146a targets TRAF6 and IRAK1 under LPS-stimulated conditions. Proteomic analysis identified 1232 proteins showing differential abundance under the predefined exploratory criteria, including proteins related to NF-κB activity, inflammasome components, nitric oxide synthesis, and IL-6-associated pathways. Proteins linked to interferon-related signaling were also altered. Targeted validation by quantitative RT-PCR and parallel reaction monitoring supported changes in selected inflammatory mediators, including PTGS2, NOS2, MAPKAPK2, and IRF3. Functionally, miR-146a overexpression was associated with reduced LPS-induced nitric oxide and IL-6 production. Together, these findings provide an exploratory proteomic overview of pathways associated with miR-146a overexpression in activated macrophages and suggest that miR-146a is associated with modulation of multiple inflammatory signaling networks under inflammatory conditions.

## 1. Introduction

Inflammation is a vital defense mechanism essential for eliminating pathogens and restoring tissue homeostasis following injury or infection [1,2]. However, unresolved or excessive inflammatory responses drive tissue damage and underlie numerous debilitating conditions, including autoimmune disorders, cardiovascular diseases, and sepsis [3,4,5]. Macrophages serve as the primary sentinels of innate immunity, initiating these inflammatory cascades upon recognizing pathogen-associated molecular patterns, such as lipopolysaccharide (LPS), via Toll-like receptor 4 (TLR4) [6,7]. While current therapeutic strategies often rely on broad immunosuppressive agents or targeted biologics neutralizing specific secreted cytokines [8], these approaches frequently induce systemic immunosuppression. Therefore, elucidating the intrinsic intracellular checkpoints that macrophages utilize to self-limit activation is of high therapeutic relevance.

MicroRNAs (miRNAs) act as crucial post-transcriptional regulators of these innate immune networks [9,10]. Among them, microRNA-146a (miR-146a) represents a classic negative feedback regulator induced directly by pro-inflammatory stimuli [11]. Upon LPS engagement, miR-146a is upregulated to dampen signaling by targeting key components of the canonical NF-κB cascade, specifically TRAF6 and IRAK1 [12,13,14]. While the direct transcript-level repression of these primary targets is well established, macrophage activation is a highly integrated process involving extensive crosstalk between parallel signaling branches, metabolic rewiring, and stress responses [15]. Whether the functional reach of miR-146a extends beyond canonical NF-κB inhibition to coordinate or buffer these broader intracellular networks remains poorly mapped at the protein level.

Global proteomics provides a powerful systems-level approach for delineating complex cellular phenotypes [16,17]. In activated macrophages, rapid transcript turnover, post-transcriptional buffering, and delayed protein degradation often create a substantial divergence between mRNA expression and actual protein abundance [18,19]. Consequently, relying solely on transcriptomic screens can obscure pathway-level alterations and downstream effector mechanisms. Quantitative mass spectrometry-based proteomic profiling bridges this gap by capturing the ultimate functional executioners of the cell, offering a more direct readout of inflammatory modulation.

In this study, we employed label-free quantitative mass spectrometry to characterize global protein abundance changes associated with miR-146a overexpression in LPS-stimulated RAW 264.7 macrophages. By comparing miR-146a mimic-transfected cells with negative control mimic-treated cells under a standardized inflammatory challenge, we aimed to identify protein networks and pathways associated with miR-146a activity. Rather than resolving the early, transient kinetics of LPS signaling, our experimental framework was specifically designed to capture downstream steady-state proteomic endpoints. Overall, this work provides an exploratory systems-level overview of the inflammatory cascades modulated by miR-146a overexpression and highlights candidate regulatory processes that may contribute to its broad anti-inflammatory effects.

## 2. Results

### 2.1. Validation of MiR-146a Mimic Overexpression and Functional Activity Under LPS-Stimulated Conditions

To validate efficient overexpression and functional activity of the miR-146a mimic under inflammatory conditions, RAW 264.7 cells were transfected with either an miR-146a mimic or a negative control mimic prior to lipopolysaccharide (LPS) stimulation. Quantitative PCR analysis confirmed a significant increase in miR-146a levels in miR-146a mimic-transfected cells compared to control mimic-transfected cells (Supplementary Figure S1a). This elevated miR-146a expression was sustained following LPS stimulation (Supplementary Figure S1b). To further assess the functional activity of the overexpressed miR-146a, we examined the mRNA levels of established miR-146a target genes within the NF-κB signaling pathway, including *Traf6* and *Irak1*. Consistent with previous reports, miR-146a overexpression was associated with significantly reduced mRNA levels of *Traf6* and *Irak1* compared to control mimic-transfected cells under LPS-stimulated conditions (Supplementary Figure S1c,d). These results confirm effective miR-146a mimic transfection and functional engagement of canonical targets, thereby validating the experimental system used for subsequent proteomic and functional analyses. As these measurements were performed at a 24 h endpoint, the present data do not exclude the possibility of transient early regulation of *Traf6* and *Irak1* after LPS stimulation. To ensure that the observed changes in the proteomic profile and inflammatory responses were not confounded by cytotoxicity, we evaluated cell viability following miR-146a mimic transfection and LPS stimulation. Cell viability assays confirmed that neither the transfection procedure nor the miR-146a overexpression induced significant cell death compared to the control mimic group (Supplementary Figure S2). These results indicate that the anti-inflammatory effects observed in our study are mediated by specific regulatory mechanisms of miR-146a rather than alterations in cell survival.

### 2.2. Global Proteomic Profiling Identifies Protein Abundance Changes Associated with MiR-146a Overexpression in LPS-Stimulated RAW 264.7 Cells

**Figure 1:**
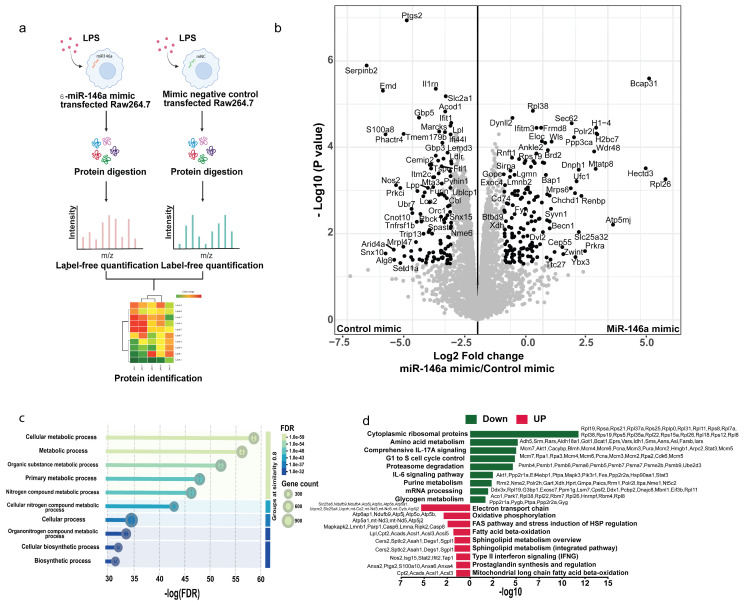
Experimental workflow and global proteomic profiling of miR-146a mimic- and control mimic-transfected RAW 264.7 cells under LPS stimulation. RAW 264.7 cells were transfected with either miR-146a mimic or control mimic and stimulated with 100 ng/mL LPS for 24 h before proteomic analysis. (**a**) Experimental workflow. (**b**) Volcano plot showing differentially abundant proteins between the two groups (*n* = 5 independent biological replicates per group). (**c**) GO Biological Process enrichment analysis. (**d**) WikiPathways enrichment analysis.

To characterize global protein abundance changes associated with miR-146a overexpression under inflammatory conditions, label-free quantitative proteomic analysis was performed in LPS-stimulated RAW 264.7 cells transfected with either an miR-146a mimic or a negative control mimic, using five independent biological replicates per group (Figure 1a). Across all samples, a total of 3915 proteins were identified. Comparative analysis identified 1232 proteins exhibiting differential abundance between the miR-146a mimic and control mimic groups (*p* < 0.05 and |log_2_ fold change| ≥ 1), including 584 proteins with increased abundance and 648 proteins with decreased abundance in the miR-146a mimic condition (Supplementary Table S1). A volcano plot illustrating these protein abundance changes is shown in Figure 1b, with 278 proteins displaying greater than twofold differences between groups.

**Figure 2:**
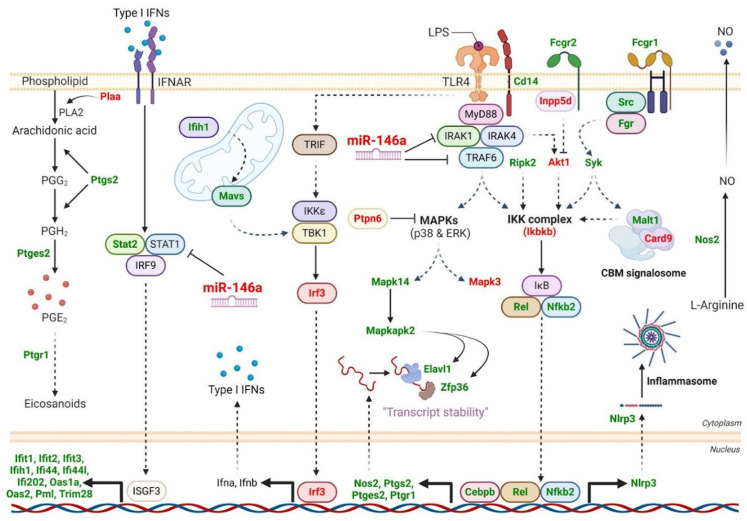
Proposed model summarizing pathways and proteins identified by proteomic and functional analyses. Proteins shown in red and green text indicate proteins with increased and decreased abundance, respectively. Created with BioRender.com.

To explore functional patterns associated with these protein abundance changes, enrichment analyses were performed using STRING. The main figure presents results from GO Biological Process and WikiPathways analyses. Biological Process enrichment analysis revealed significant enrichment of metabolism-related biological processes, consistent with previous reports describing broad metabolic remodeling during inflammatory responses (Figure 1c). WikiPathways analysis, conducted separately for proteins with increased and decreased abundance, demonstrated that proteins with decreased abundance were associated with pathways including ribosomal function, amino acid metabolism, IL-17A signaling, cell cycle regulation, proteasome-mediated degradation, IL-6-associated signaling, and mRNA processing. In contrast, proteins with increased abundance were enriched in pathways related to mitochondrial metabolism, oxidative phosphorylation, fatty acid β-oxidation, sphingolipid metabolism, interferon-associated signaling, and prostaglandin-related processes (Figure 1d). Examination of individual proteins revealed altered abundance of multiple components linked to inflammatory signaling networks. Several transcription factors and signaling molecules associated with NF-κB-related pathways, including REL, NFKB2, and CEBPB, exhibited reduced abundance in the miR-146a mimic condition, accompanied by decreased abundance of inflammatory mediators such as NLRP3, NOS2, and PTGS2. In addition, altered abundance of upstream signaling molecules, including kinases involved in immune receptor signaling, was observed. Conversely, increased abundance of the phosphatase INPP5D, a known negative regulator of inflammatory signaling, was detected. Proteomic analysis also identified altered abundance of proteins involved in interferon-associated pathways, including STAT2 and selected interferon-stimulated proteins. These findings suggest an association between miR-146a overexpression and modulation of interferon-related protein networks under LPS-stimulated conditions. However, whether these changes reflect transcriptional, post-transcriptional, or signaling-level regulation cannot be determined from proteomic data alone. A schematic summary of pathways and protein networks identified by the proteomic and validation analyses is presented as a proposed model in Figure 2.

### 2.3. Assessment of mRNA Abundance of Selected Inflammatory Mediators by Quantitative RT-PCR

**Figure 3:**
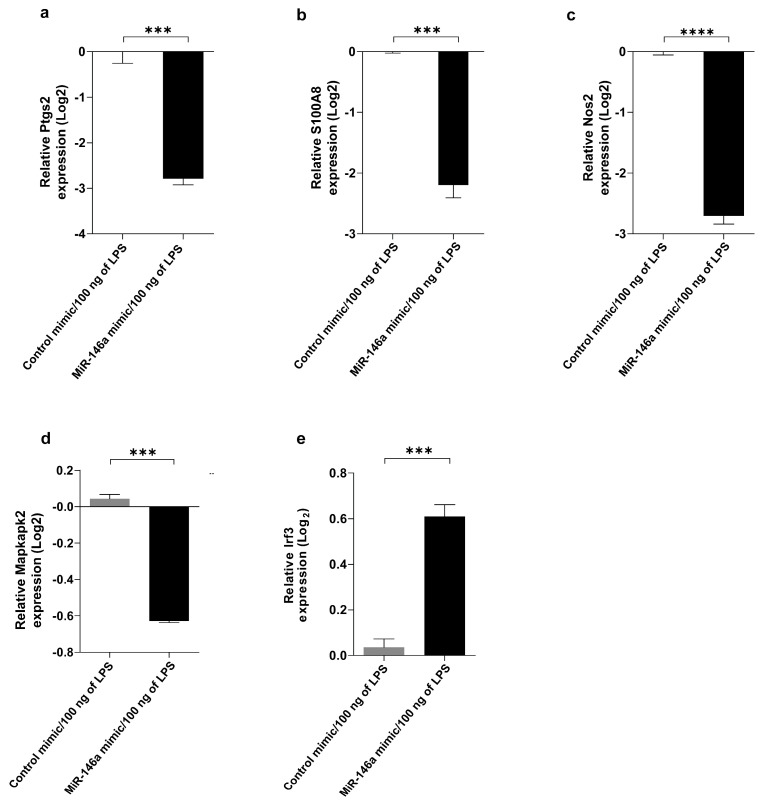
RAW 264.7 cells were transfected with either miR-146a mimic or control mimic and subsequently stimulated with 100 ng/mL LPS for 24 h. The mRNA abundance of Ptgs2 (**a**), S100a8 (**b**), Nos2 (**c**), Mapkapk2 (**d**), and Irf3 (**e**) was assessed by qRT-PCR. Relative gene expression was normalized to β-actin and expressed relative to the control mimic + 100 ng/mL LPS group. Data are presented as mean ± SEM (*n* = 3 independent biological replicates per group). Statistical significance was assessed using an unpaired two-tailed Student’s *t*-test. *** *p* < 0.001 and **** *p* < 0.0001.

To further examine whether protein abundance changes identified by proteomic analysis were accompanied by corresponding changes at the mRNA level, quantitative RT-PCR was performed to assess the mRNA abundance of selected genes, including *Ptgs2*, *S100a8*, *Nos2*, *Mapkapk2*, and *Irf3*. These genes were selected based on their established roles in LPS-induced inflammatory responses, their altered protein abundance in the proteomic dataset, and their representation of distinct inflammatory regulatory axes. Quantitative RT-PCR analysis revealed significantly reduced mRNA abundance of *Ptgs2* (*p* = 0.007), *S100a8* (*p* = 0.001), *Nos2* (*p* < 0.0001), and *Mapkapk2 (p* = 0.0007) in RAW 264.7 cells transfected with the miR-146a mimic compared to control mimic-transfected cells following LPS stimulation (Figure 3). In contrast, *Irf3* mRNA abundance was significantly increased under the same conditions (*p* = 0.0012). These findings demonstrate concordant changes in mRNA abundance for selected inflammatory mediators and suggest that miR-146a overexpression is associated with coordinated regulation at both transcript and protein abundance levels under inflammatory conditions. However, the present data do not distinguish whether these changes arise from transcriptional or post-transcriptional regulatory mechanisms.

### 2.4. Targeted Peptide-Level Validation of Selected Proteins by Parallel Reaction Monitoring

**Figure 4:**
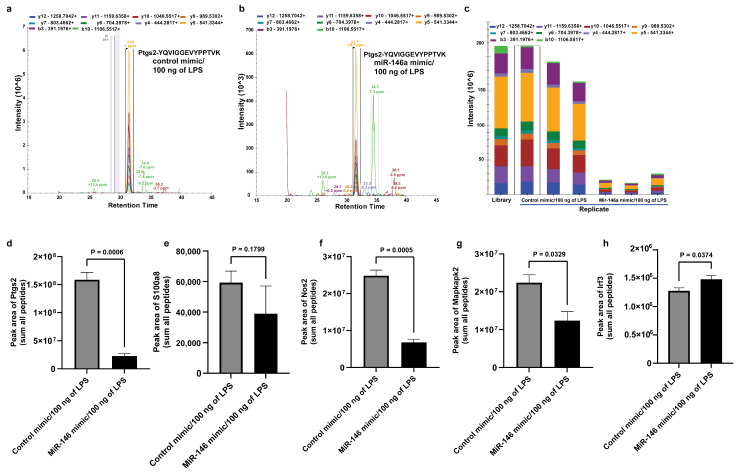
PRM-based peptide-level validation of selected proteins associated with miR-146a overexpression in LPS-stimulated RAW 264.7 cells. Representative PRM data for the PTGS2 peptide YQVIGGEVYPPTVK are shown in (**a**–**c**), including extracted ion chromatograms from control mimic/LPS and miR-146a mimic/LPS samples (**a**,**b**) and the corresponding transition/replicate peak profile (**c**). Quantitative PRM results for PTGS2 (**d**), S100A8 (**e**), NOS2 (**f**), MAPKAPK2 (**g**), and IRF3 (**h**) are shown as summed peak areas of all monitored peptides. PTGS2, NOS2, and MAPKAPK2 were significantly decreased, whereas IRF3 was significantly increased, in miR-146a mimic-transfected cells relative to control mimic-transfected cells following LPS stimulation. S100A8 showed a decreasing trend that did not reach statistical significance. Data are presented as mean ± SEM from 3 independent biological replicates. Statistical significance was assessed using an unpaired two-tailed Student’s *t*-test.

To further assess peptide-level abundance changes in selected proteins identified in the global proteomic analysis, targeted parallel reaction monitoring (PRM) was performed for PTGS2, S100A8, NOS2, MAPKAPK2, and IRF3. Representative PRM data for the PTGS2 peptide YQVIGGEVYPPTVK are shown in Figure 4a–c, including extracted ion chromatograms from the control mimic/LPS and miR-146a mimic/LPS conditions (Figure 4a,b) and the corresponding transition/replicate peak profile (Figure 4c). These representative traces show a visibly lower PRM signal in the miR-146a mimic condition than in the control mimic condition. Quantitative PRM analysis demonstrated peptide-level abundance patterns that were largely consistent with the global proteomic dataset. Specifically, PTGS2, NOS2, and MAPKAPK2 showed significantly lower peptide abundance in miR-146a mimic-transfected cells than in control mimic-transfected cells following LPS stimulation (Figure 4d, Figure 4f and Figure 4g, respectively). IRF3-derived peptides showed a modest but significant increase in abundance in the miR-146a mimic condition (Figure 4h). In contrast, S100A8-derived peptides showed a decrease in the same direction, but this change did not reach statistical significance in the PRM analysis (Figure 4e). Overall, these targeted PRM results support the direction of change observed in the discovery proteomics dataset, while also indicating that not all candidates were validated with equal statistical strength at the peptide level. Because PRM provides targeted quantification of selected peptides rather than direct measurement of total protein abundance or protein activity, these findings should be interpreted as supportive orthogonal validation of the discovery proteomics results, rather than definitive confirmation of total protein-level regulation.

### 2.5. MiR-146a Overexpression Is Associated with Reduced Nitric Oxide and IL-6 Production in LPS-Stimulated Macrophages

**Figure 5:**
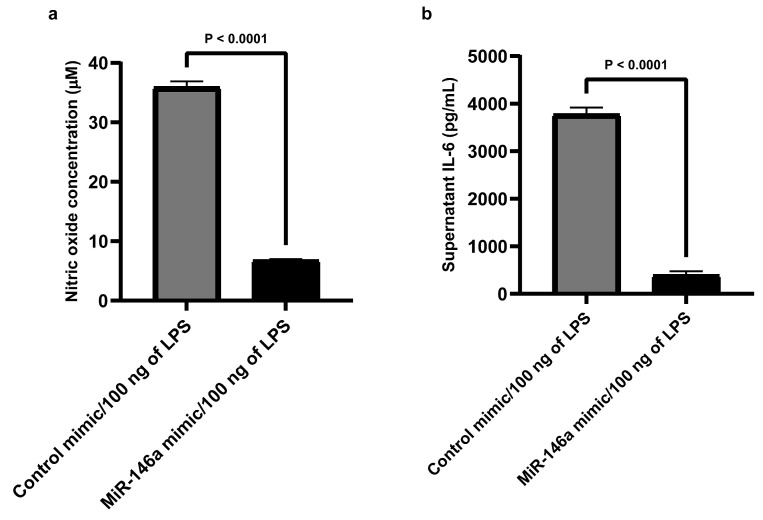
Reduced nitric oxide and IL-6 production in miR-146a mimic-transfected RAW 264.7 cells under LPS stimulation. Nitric oxide concentration (**a**) and IL-6 concentration (**b**) were measured in culture supernatants from control mimic- and miR-146a mimic-transfected RAW 264.7 cells after stimulation with 100 ng/mL LPS for 24 h. Data are presented as mean ± SEM from 3 independent biological replicates. Statistical significance was assessed using an unpaired two-tailed Student’s *t*-test.

To assess functional inflammatory outputs associated with miR-146a overexpression, nitric oxide (NO) and interleukin-6 (IL-6) production were measured in LPS-stimulated RAW 264.7 macrophages transfected with either an miR-146a mimic or a negative control mimic. Under the fixed LPS challenge, miR-146a mimic-transfected cells showed significantly lower NO and IL-6 levels than control mimic-transfected cells (Figure 5). IL-6 was not detected in the cell-lysate-based proteomic analysis, likely reflecting its low intracellular abundance and rapid secretion, which limits detection by whole-cell LC-MS/MS. Together, these results indicate that miR-146a overexpression is associated with attenuation of key inflammatory outputs under LPS-stimulated conditions.

## 3. Discussion

This study aimed to characterize global protein abundance changes associated with the anti-inflammatory effects of miR-146a in LPS-stimulated RAW 264.7 macrophages using quantitative proteomics. The present study used a fixed LPS stimulation condition and was not designed to determine the optimal LPS dose or to define the temporal kinetics of inflammatory signaling. Therefore, the findings should be interpreted as endpoint-associated proteomic and functional changes under the selected experimental condition. Our initial findings confirmed the efficient overexpression of miR-146a mimic and the subsequent downregulation of its established targets, Traf6 and Irak1, at the mRNA level, consistent with previous reports [20]. This successful manipulation allowed us to investigate the broader impact of miR-146a on the macrophage proteome during an inflammatory challenge. An important limitation of the present study is that only two LPS-treated groups were compared. Inclusion of additional groups, such as miR-146a mimic without LPS, control mimic without LPS, or LPS-only conditions, would improve resolution of basal, transfection-related, and LPS-dependent effects and should be incorporated in future studies.

Our global proteomic analysis revealed a substantial number of differentially expressed proteins (DEPs) in miR-146a mimic-transfected RAW 264.7 cells compared to the control mimic group following LPS stimulation. Previous studies have shown that miR-146a suppresses TNF, IL-1β, and IL-6 expression in THP-1 monocytes and contributes to endotoxin tolerance-like responses in RAW 264.7 macrophages [20,21,22,23]. While the impact of miR-146a overexpression on protein changes is shown in this paper, the mechanisms underlying these effects remain largely unknown. Our proteomic analysis showed reduced abundance of transcription factors associated with LPS-induced NF-κB signaling, including REL, NFKB2, and CEBPB. These changes were accompanied by reduced abundance of inflammatory mediators at the protein level, including NLRP3, NOS2, and prostaglandin-associated enzymes such as PTGS2, PTGES2, and PTGR1. Moreover, our proteomic data also indicated a downregulation of Stat2, a key component of the IFN-mediated JAK-STAT pathway, alongside the downregulation of several interferon-stimulated genes (ISGs) [24,25,26]. These observations suggest that miR-146a overexpression is associated with alterations in interferon-related protein networks. However, the mechanistic basis and functional significance of these changes remain unclear and require further investigation. Importantly, the broader differentially abundant proteins identified in this study should not be interpreted as direct miR-146a targets without additional target-prediction and functional validation.

Our targeted qRT-PCR validation of *Ptgs2*, *S100a8*, *Nos2*, *Mapkapk2*, and *Irf3* transcripts largely mirrored the proteomic trends, supporting the internal consistency of the mass-spectrometry dataset. Although Western blot analysis was not performed in the present study to further validate protein expression, the biological mechanism of miR-146a is robustly supported by the multi-layered consistency of our findings. We observed a concordant downregulation of inflammatory mediators, including *PTGS2*, *NOS2*, *MAPKAPK2*, and *IRF3*, at both the transcriptional level (via qRT-PCR, Figure 3) and the protein level (via discovery proteomics and targeted PRM analysis) [27]. This multi-omics approach—demonstrating that miR-146a overexpression leads to a simultaneous reduction in both mRNA and protein abundance—provides strong evidence of miR-146a-mediated post-transcriptional silencing. Furthermore, our validation of the canonical upstream signaling components, *Traf6* and *Irak1* (Figure S1), provides a clear mechanistic basis for the observed dampening of the NF-κB inflammatory signaling pathway.

In line with previous studies identifying COX-2 as an miR-146a-5p target [28,29] and demonstrating suppression of NF-κB-dependent inflammatory mediators, including COX-2, iNOS, and nitric oxide, in inflammatory cell models [30,31,32], we observed marked downregulation of Ptgs2 and Nos2 mRNAs. These findings are consistent with the reduced NO and IL-6 production observed in functional assays. Although PRM analysis supported peptide-level abundance changes for several targets, S100A8 did not reach statistical significance [33]. This variability is consistent with prior reports describing context-dependent regulation of S100A8 by miR-146a in intestinal inflammation models, where both miR-146a overexpression and inhibition have been associated with altered S100A8 expression [33]. The concurrent reduction of NO and IL-6 production, together with decreased Nos2 transcript abundance and altered IL-6-associated pathway proteins, suggests coordinated attenuation of inflammatory outputs in miR-146a-overexpressing macrophages. However, the present data do not establish direct mechanistic links between these mediators. Collectively, these findings place miR-146a within a broader regulatory network modulating inflammatory responses downstream of Toll-like receptor activation, consistent with previous systems-level and regulatory studies [22,34,35].

In summary, our integrated proteomic analyses suggest that miR-146a is associated with broader inflammatory modulation beyond canonical NF-κB signaling. Protein abundance changes related to IL-6-associated pathways, interferon responses, and nitric oxide and prostaglandin biosynthesis were observed in association with miR-146a overexpression, suggesting coordinated attenuation of macrophage activation. These results expand the systems-level view of inflammatory pathways associated with miR-146a overexpression under inflammatory conditions and identify candidate pathways for future mechanistic investigation. Further work will be needed to define the molecular basis of these associations and to validate their relevance in primary macrophages and in vivo models.

## 4. Limitations

While this study provides a broad exploratory proteomic landscape of the anti-inflammatory networks associated with miR-146a, several limitations must be noted. First, to maintain a focused comparative design, our experimental model utilized only two LPS-stimulated groups. Future studies incorporating additional control groups—such as miR-146a mimic-only, negative control mimic-only, and LPS-only conditions—would provide a higher-resolution dissection of basal, transfection-related, and LPS-specific effects. Second, the use of a single 24 h endpoint precludes the resolution of early, transient signaling events (such as initial TRAF6 and IRAK1 degradation kinetics) and dynamic receptor–ligand interactions. Finally, while the RAW 264.7 cell line serves as a robust in vitro discovery model, it does not fully recapitulate the complex biology of primary macrophages or physiological in vivo environments. Future investigations utilizing primary cells (e.g., bone marrow-derived macrophages or peritoneal macrophages), comprehensive time-course analyses, and in vivo inflammatory models are necessary to further clinically and physiologically validate these exploratory findings.

## 5. Materials and Methods

### 5.1. Cell Culture

The murine macrophage cell line RAW 264.7 obtained from the American Type Culture Collection (ATCC, Manassas, VA, USA) was cultured in high-glucose Dulbecco’s Modified Eagle Medium (DMEM) supplemented with 10% heat-inactivated fetal bovine serum (FBS) and 1% penicillin–streptomycin, and maintained at 37 °C in a humidified atmosphere containing 5% CO_2_. Cells were seeded in 100 mm culture dishes at a density of 2.2 × 10^6^ cells per dish and incubated for 24 h prior to transfection. The number of biological replicates depended on the downstream assay. Global proteomic analysis was performed using five independent biological replicates per group, whereas qRT-PCR, PRM, nitric oxide, and IL-6 assays were performed using three independent biological replicates per group.

### 5.2. Transfection

RAW 264.7 cells were transfected with either 40 nM miR-146a mimic or 40 nM negative control mimic (Gibthai Co., Ltd., Bangkok, Thailand) using Lipofectamine^®^ RNAiMAX (Invitrogen, Thermo Fisher Scientific, Waltham, MA, USA) according to the manufacturer’s protocol. After 24 h of transfection, cells were stimulated with 100 ng/mL lipopolysaccharide (LPS) in serum-free DMEM for an additional 24 h before harvesting cells and culture supernatants. A fixed stimulation condition of 100 ng/mL LPS for 24 h was selected based on established protocols to provide a standardized inflammatory challenge. This specific condition was chosen to capture downstream functional and global proteomic endpoints, facilitating a direct comparison between miR-146a overexpression and control conditions. The experimental design intentionally focused on the steady-state inflammatory outcome rather than profiling the early temporal kinetics or dose-responsiveness of LPS signaling.

### 5.3. Cell Viability Assay

To assess the potential cytotoxicity of the miR-146a mimic transfection, cell viability was evaluated using the 3-(4,5-dimethylthiazol-2-yl)-2,5-diphenyltetrazolium bromide (MTT) assay (Invitrogen, Thermo Fisher Scientific). Briefly, RAW 264.7 cells were seeded in 96-well plates and subjected to the same transfection and LPS stimulation protocols as the main experiments. Following the treatment period, MTT reagent was added to each well, and the cells were incubated for an additional 2–4 h. The resulting formazan crystals were dissolved in dimethyl sulfoxide (DMSO), and the absorbance was measured at 570 nm using a microplate reader. Cell viability was expressed as a percentage relative to the control + LPS group.

### 5.4. miRNA and Total RNA Extraction

Total miRNA was isolated using the miRNeasy^®^ Mini Kit (Qiagen, Hilden, Germany) according to the manufacturer’s instructions. Briefly, samples were lysed in 700 µL of QIAzol Lysis Reagent. To monitor extraction efficiency and for subsequent normalization, 3.5 µL of synthetic Caenorhabditis elegans cel-miR-39 (4 × 10^9^ copies/µL) was added to each sample after lysis and prior to chloroform extraction as an external spike-in control. Purified miRNA was eluted in 30 µL of RNase-free water. For mRNA analysis, total RNA was extracted separately using the RNeasy^®^ Mini Kit (Qiagen). RNA quantity and purity were assessed using a NanoDrop^®^ 2000 spectrophotometer (Thermo Fisher Scientific, Waltham, MA, USA).

### 5.5. Reverse Transcription and Quantitative PCR

For miR-146a expression analysis, cDNA was synthesized from 250 ng miRNA using the TaqMan™ Reverse Transcription Kit (Applied Biosystems, Thermo Fisher Scientific, Waltham, MA, USA) according to the manufacturer’s instructions. Quantitative PCR was performed on a QuantStudio™ 5 Real-Time PCR System (Applied Biosystems, Thermo Fisher Scientific, Waltham, MA, USA) in a 20 µL reaction containing 10 µL of TaqMan™ Universal PCR Master Mix, 1 µL of TaqMan™ MicroRNA Assay, 6 µL of RNase-free water, and 3 µL of cDNA. The PCR cycling conditions consisted of an initial incubation at 50 °C for 2 min and 95 °C for 10 min, followed by 50 cycles of 95 °C for 15 s and 60 °C for 1 min. miR-146a expression levels were normalized to the external spike-in control cel-miR-39 and calculated using the 2^^−ΔΔCt^ method (where ΔCt = Ct_target − Ct_Spike-in and ΔΔCt = ΔCt_target − mean of ΔCt baseline) [36]. Data are presented as Log2 relative expression.

For mRNA analysis, cDNA was synthesized from 250 ng of total RNA using a reverse transcription kit containing MultiScribe™ Reverse Transcriptase, random hexamers, dNTPs, RNase inhibitor, MgCl_2_, and 10× RT buffer (Applied Biosystems, Thermo Fisher Scientific, Waltham, MA, USA). Quantitative PCR was performed using PowerUp™ SYBR™ Green Master Mix (Applied Biosystems, Thermo Fisher Scientific, Waltham, MA, USA) on a QuantStudio™ 5 Real-Time PCR System (Applied Biosystems, Thermo Fisher Scientific, Waltham, MA, USA). Gene expression levels of PTGS2, S100A8, NOS2, MAPKAPK2, IRF3, TRAF6, and IRAK1 were normalized to β-actin, and relative expression levels were calculated using the 2^^−ΔΔCt^ method, (where ΔCt = Ct_target − Ct_β-actin and ΔΔCt = ΔCt_target − mean of ΔCt_baseline) [36]. Data are presented as Log2 relative expression [37,38].

Primer sequences are listed in Table 1. All qRT-PCR analyses were performed using three independent biological replicates per group.

### 5.6. Sample Preparation and LC-MS/MS

After treatment, RAW 264.7 cells were washed with phosphate-buffered saline (PBS) and lysed in 8 M urea prepared in 50 mM Tris-HCl (pH 8.0). Protein concentration was determined using a BCA assay (Thermo Fisher Scientific, Waltham, MA, USA). For each sample, 100 µg of total protein was processed using the Thermo Scientific EasyPep Mini MS Sample Prep Kit (Cat. No. A40006, Rockford, IL, USA) according to the manufacturer’s instructions. The resulting peptides were analyzed using an EASY-nLC 1000 system coupled to a Q Exactive Plus Orbitrap mass spectrometer (Thermo Fisher Scientific, Bremen, Germany) equipped with a nano-electrospray ion source at a flow rate of 300 nL/min, followed by a linear acetonitrile gradient (5–40% for 60 min, followed by 40–95% for 30 min). Raw data were processed using MSFragger (version 2.2) within FragPipe (version 12.1) against the mouse protein database (17,224 entries) with decoy sequences appended. MS/MS searches were performed using a 10 ppm main search tolerance and a 0.2 Da first search tolerance, with carbamidomethylation set as a fixed modification and methionine oxidation and protein N-terminal acetylation set as variable modifications.

### 5.7. Label-Free Quantification and Statistical Analysis of Proteomics Data

Peptide-spectrum matches, peptides, and proteins were filtered at 1% false discovery rate (FDR) using a target–decoy strategy as implemented in FragPipe/MSFragger [39]. Label-free quantification (LFQ) was performed. Protein intensity values were log2-transformed prior to statistical analysis. Proteins quantified in at least three out of five biological replicates per group were retained for analysis. Missing values were imputed in FragPipe prior to statistical testing. Differential protein abundance was assessed using unpaired *t*-tests. Given the exploratory nature of global proteomic profiling, *p*-values (*p* < 0.05) were used to nominate candidate differentially abundant proteins for downstream validation by qRT-PCR and PRM. The mass spectrometry proteomics data have been deposited to the ProteomeXchange Consortium via the PRIDE partner repository with the dataset identifier PXD064135 [40].

### 5.8. Pathway Enrichment Analysis

Enrichment analysis was performed using STRING (09/2025) with Gene Ontology Biological Process (GO BP), KEGG, and WikiPathways databases. Results shown in the main figure correspond to GO Biological Process and WikiPathways analyses. Differentially abundant proteins were separated into increased and decreased abundance sets and analyzed using default STRING settings, using the full identified protein list as background [41].

### 5.9. Parallel Reaction Monitoring (PRM)

Briefly, 100 ng of peptides was loaded onto a 100 µm × 50 mm trap column and separated by an analytical C18 column (75 µm × 200 mm, 3 µm) with a flow rate of 300 nL/min, followed by a linear acetonitrile gradient (5–40% for 60 min, 40–95% for 30 min). The MS/MS scan was performed using the following settings: isolation window, 2 *m*/*z*; automatic gain control target, 2 × 10^5^; maximum injection time, 130 ms; and normalized collision energy, 27 eV. The PRM raw data were analyzed with Skyline (version 3.5.0) with manual detection [42]. Precursors were confidently detected when Skyline auto-detected peaks met related requirements, including a library dot product > 0.6, and an isotope dot product > 0.6. Three consecutive ions in the most abundant peptides were used for quantification. The chromatographic peak and raw peak area of the target peptides were exported as the sum of peptides. The peak areas of target peptides were calculated relative to the control mimic group for comparison with the miR-146a mimic group. PRM analysis was performed using three independent biological replicates per group.

### 5.10. IL-6 Measurement by Using ELISA

The levels of interleukin-6 (IL-6) in cell culture supernatants were measured using an ELISA kit (Thermo Fisher Scientific) according to the manufacturer’s guidelines. In summary, mouse IL-6 standards and the collected supernatants were added to a 96-well ELISA plate and incubated at room temperature for 2 h. After incubation, the liquid was aspirated, and the wells were washed five times with 1× PBS containing 0.05% Tween-20. Next, a biotin-conjugated anti-mouse IL-6 antibody (diluted 1:249) was then added and incubated for another 1 h at room temperature. The wells were subsequently aspirated, washed, and dried using absorbent paper. Following this, 50 µL of diluted Avidin-HRP was added to each well and incubated for 30 min. After repeating the wash and drying steps, tetramethylbenzidine (TMB) substrate solution was added to each well, and the plate was incubated for 15 min in the dark. The reaction was stopped by adding 30 µL of stop solution to each well. Absorbance was measured at 450 nm using a microplate reader. The samples were measured in triplicate, and the concentrations of IL-6 were calculated using a standard curve and adjusted based on the dilution factor. IL-6 measurements were performed using three independent biological replicates per group.

### 5.11. Nitric Oxide Assay

RAW 264.7 cells were treated with 100 ng/mL LPS (Sigma-Aldrich, St. Louis, MO, USA) for 24 h following the transfection with either the miR-146a mimic or the negative control mimic. After treatment, cell culture supernatants were collected and analyzed for nitric oxide (NO) concentration. Briefly, a standard curve was generated using sodium nitrite standards prepared in DMEM. Standard solutions or cell supernatants were mixed with 1% sulfanilamide in 5% phosphoric acid and incubated in the dark for 10 min in a 96-well plate. Subsequently, 0.1% N-1-naphthyl ethylenediamine dihydrochloride (NED) was added, followed by a 10 min incubation at room temperature in the dark. Absorbance was then immediately measured at 540 nm using a microplate reader. All samples were assayed in triplicate, and NO concentrations were calculated using a standard curve. Nitric oxide measurements were performed using three independent biological replicates per group.

### 5.12. Statistical Analysis

The normal distribution of the data was assessed using the Shapiro–Wilk test. For comparisons between two groups, statistical significance was determined using an unpaired two-tailed Student’s *t*-test in GraphPad Prism 11.0 (GraphPad Software, Boston, MA, USA). Data are presented as mean ± SEM, and *p* < 0.05 was considered statistically significant.

## 6. Conclusions

Overall, our findings demonstrate that the anti-inflammatory reach of miR-146a extends well beyond its canonical role in suppressing NF-κB signaling. By taking a system-wide proteomic approach, we observed a coordinated dampening of macrophage activation following LPS stimulation. This included clear reductions in pathways driving nitric oxide and prostaglandin biosynthesis, as well as IL-6 and interferon-related responses. Ultimately, this dataset serves as a valuable resource, pointing to novel regulatory mechanisms that can now be dissected in more complex biological settings, such as primary macrophages and in vivo disease models.

## Figures and Tables

**Table 1:** List of primer sequences.

Primer Name		Sequence
**PTGS2**	Forward	5′-TGAGTACCGCAAACGCTTCT-3′
	Reverse	5′-ACGAGGTTTTTCCACCAGCA-3′
**S100a8**	Forward	5′-GGAGTTCCTTGCGATGGTGA-3′
	Reverse	5′-GGCCAGAAGCTCTGCTACTC-3′
**NOS2**	Forward	5′-GGCAGCCTGTGAGACCTTTG-3′
	Reverse	5′-GCATTGGAAGTGAAGCGTTTC-3′
**MAPKAPK2**	Forward	5′-GGATATTTGGTCCGTGGGCT-3′
	Reverse	5′-CCTGGGGTTCCAACGAGTCT-3′
**IRF3**	Forward	5′-GCCCCAATGTGAACAACTTC-3′
	Reverse	5′-ACCTCGAACTCCCATTGTTC-3′
**TRAF6**	Forward	5′-AGGAGATCCAGGGCTACGAT-3′
	Reverse	5′-TGCATCCCTTATGGATTTGA-3′
**IRAK1**	Forward	5′-CGCTTCTACAAAGTGATGGAC-3′
	Reverse	5′-TGTGAACGAGGTCAGCTACG-3′
**Beta-actin**	Forward	5′-AGAGGGAAATCGTGCGTGAC-3′
	Reverse	5′-CAATAGTGATGACCTGGCCGT-3′

## Data Availability

In addition to the PRIDE deposition for the mass spectrometry proteomics data (PXD064135), the minimal underlying dataset required to reproduce the reported figures and summary statistics is provided in S1 Dataset. These data include the numerical values underlying Figure S1, Figure 3, Figure 4 and Figure 5, including qRT-PCR, PRM, nitric oxide, and IL-6 analyses.

## Supplementary Materials

The following supporting information can be downloaded at: https://www.mdpi.com/article/10.3390/ijms27146514/s1.
