# Supplementary figures and images for: Global Proteomic Analysis Reveals Inflammatory Pathway Modulation Associated with miR-146a in LPS-Stimulated Macrophages
Source: Int J Mol Sci. 2026 Jul 22;27(14):6514. doi: 10.3390/ijms27146514 (PMC13411526; doi:10.3390/ijms27146514)

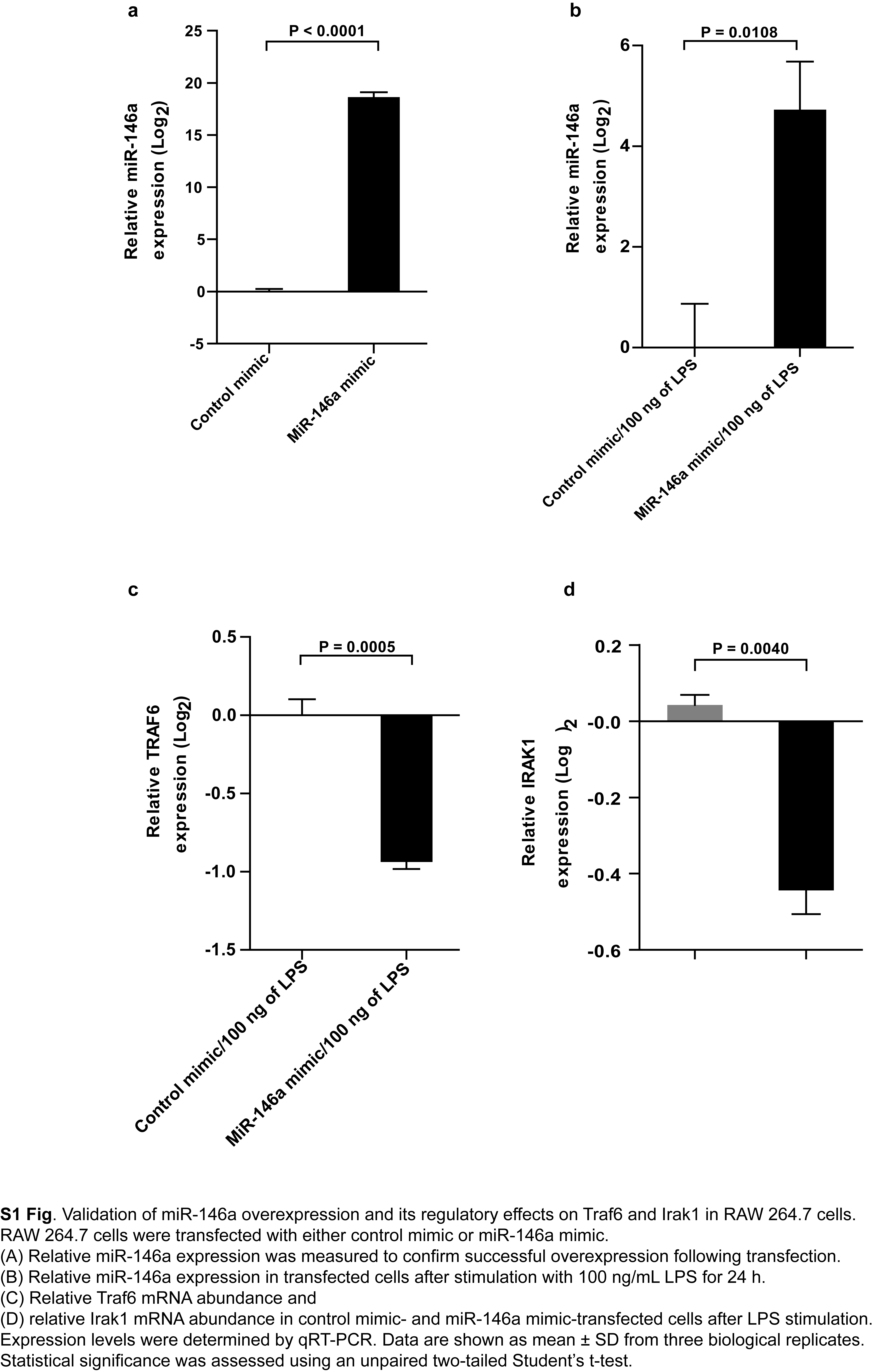

Supplement: Supplementary file 1 [file ijms-27-06514-s001.zip › Supplement Figure S1.png]

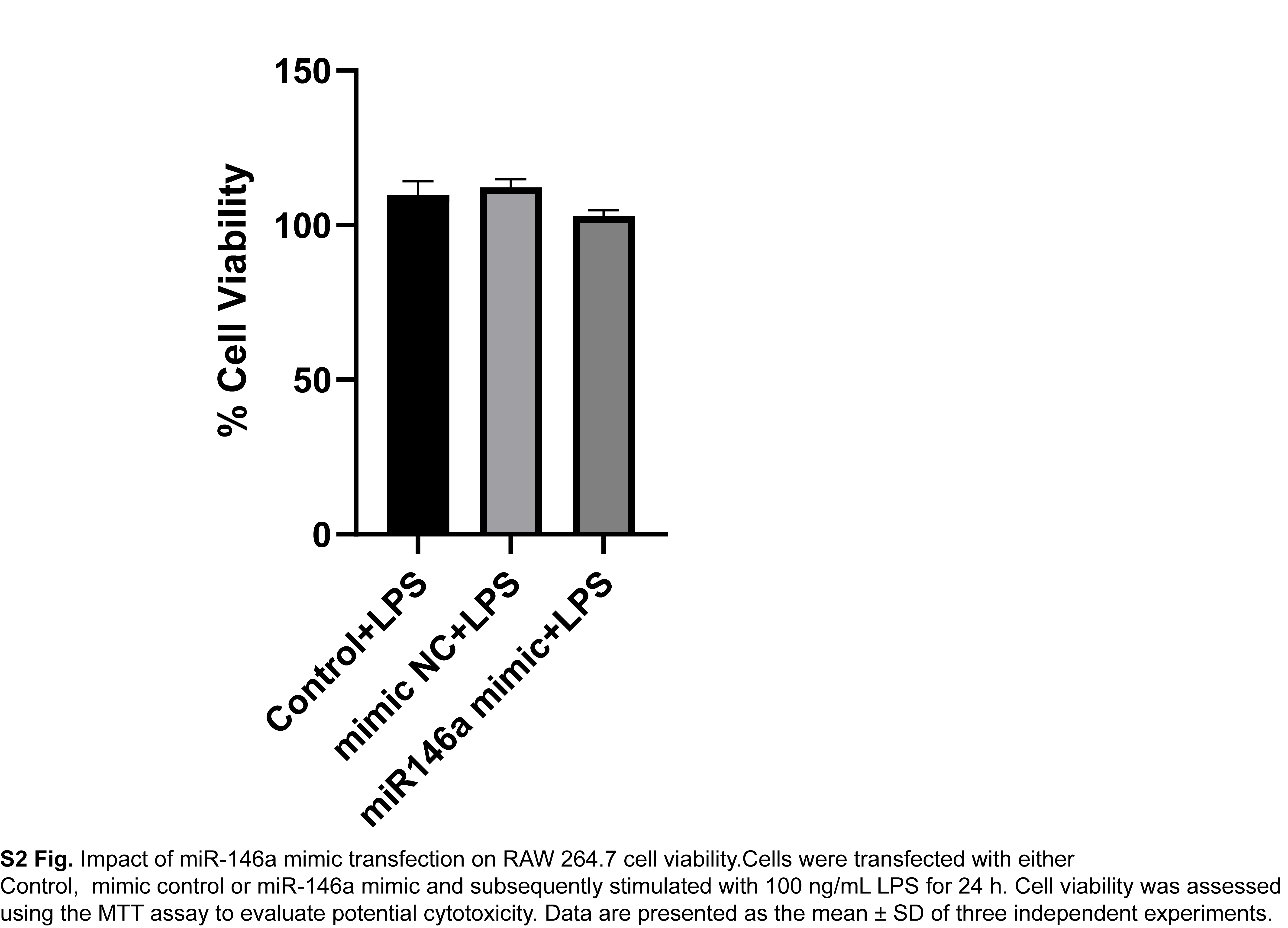

Supplement: Supplementary file 1 [file ijms-27-06514-s001.zip › Supplement Figure S2.png]
